# Exploring the Limits of Cyanobactin Macrocyclase PatGmac: Cyclization of PawS-Derived Peptide Sunflower Trypsin Inhibitor-1 and Cyclotide Kalata B1

**DOI:** 10.1021/acs.jnatprod.2c01158

**Published:** 2023-03-14

**Authors:** Taj Muhammad, Wael E Houssen, Louise Thomas, Cristina-Nicoleta Alexandru-Crivac, Sunithi Gunasekera, Marcel Jaspars, Ulf Göransson

**Affiliations:** † Pharmacognosy, Department of Pharmaceutical Biosciences, Uppsala University, Biomedical Centre, Box 591, SE-75124 Uppsala, Sweden; ‡ Department of Chemistry, Marine Biodiscovery Centre, University of Aberdeen, Aberdeen AB24 3UE, Scotland, U.K.; § Institute of Medical Sciences, University of Aberdeen, Aberdeen AB25 2ZD, Scotland, U.K.

## Abstract

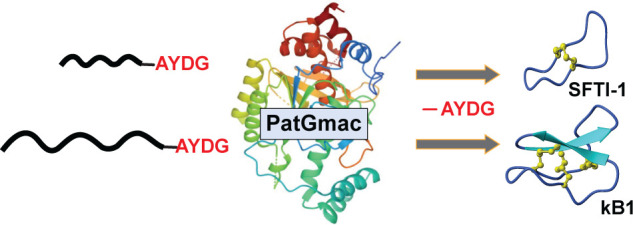

The subtilisin-like macrocyclase PatGmac is produced by the marine cyanobacterium *Prochloron didemni*. This enzyme is involved in the last step of the biosynthesis of patellamides, a cyanobactin type of ribosomally expressed and post-translationally modified cyclic peptides. PatGmac recognizes, cleaves, and cyclizes precursor peptides after a specific recognition motif comprised of a C-terminal tail with the sequence motif -AYDG. The result is the native macrocyclic patellamide, which has eight amino acid residues. Macrocyclase activity can be exploited by incorporating that motif in other short linear peptide precursors, which then are formed into head-to-tail cyclized peptides. Here, we explore the possibility of using PatGmac in the cyclization of peptides larger than the patellamides, namely, the PawS-derived peptide sunflower trypsin inhibitor-1 (SFTI-1) and the cyclotide kalata B1. These peptides fall under two distinct families of disulfide constrained macrocyclic plant peptides. They are both implicated as scaffolds for drug design due to their structures and unusual stability. We show that PatGmac can be used to efficiently cyclize the 14 amino acid residue long SFTI-1, but less so the 29 amino acid residue long kalata B1.

Nature is an infinite source of inspiration for drug discovery and biotechnology. Recent examples include naturally occurring head-to-tail cyclic peptides, including PawS-derived peptides (PDPs) and cyclotides that attract interest because of their chemical, physical, and biological stability.^1−3^ However, creating macrocyclic peptides is still challenging. One way to meet this challenge is to again turn to nature and exploit the biosynthetic machinery of peptide cyclizing enzymes. In this work, we explore the limits of one such enzyme, patellamide G macrocyclase (PatGmac), for the production of larger peptide macrocycles.

PatGmac is a subtilisin-like macrocyclase produced by the cyanobacterium *Prochloron didemni*, a symbiont of the sea squirt *Lissoclinum patella*.^4^ As one of the seven genes that form the patellamide gene cluster, PatG possesses a subtilisin-like domain referred to as PatGmac that has macrocyclase activity in the patellamide biosynthesis pathway. PatGmac recognizes C-terminal signature positions P1′–P4′, comprised of the sequence AYDG.^4^ The catalytic serine in PatGmac forms an acyl complex with the peptide substrate. The recognition signals AYDG bind to the helix-turn-helix macrocyclization motif of the enzyme, shielding the acyl-enzyme intermediate from water. The P1 proline adopts a *cis* confirmation, which results in the peptide bending back on itself, allowing the N-terminus to attack the acyl complex, resulting in cyclization.^4,5^ In *P. didemni*, PatG is involved in the last step of the production of patellamides from a 71-residue precursor protein, PatE. Final patellamides are six to eight amino acid residue cyclic peptides and contain oxazoline and thiazoline groups derived from heterocyclization of serine, threonine, or cysteine. But can PatGmac also cyclize larger peptides?

To date, cyclic peptides have been isolated from microorganisms, plants, and animals. Cyclotides and PawS-derived peptides are two distinct families of cyclic peptides, with kalata B1 (kB1) and SFTI-1 being prototypes of the respective peptide families.^3,6−8^ These peptides have attracted great interest because of their unique topology, stability, and applicability as scaffolds in peptide drug design and engineering.^2,9,10^ The two peptide families present different sizes and different properties for drug design: SFTI-1 is a 14-residue long backbone cyclized peptide from the PDP family. It contains a double-stranded antiparallel β-sheet connected via a single disulfide bond between Cys3 and Cys11.^11^ Cyclotides comprise a large family of plant-derived peptides of approximately 30 amino acid residues, which is characterized by a cyclic backbone combined with three conserved disulfide bonds forming a cyclic cystine knot (CCK) motif.^1,2,12^

Despite the current knowledge of the *in vivo* processing and biosynthesis of cyclotides and SFTI-1, opening a window of opportunity for their transgenic expression, the most robust approach of production is still by chemical synthesis. Currently, cyclotides and SFTI-1 peptides are produced by solid phase peptide synthesis (SPPS, Boc or Fmoc based) in combination with native chemical ligation (NCL) to form the head-to-tail cyclic backbone.^13−15^ As an alternative to NCL, the direct use of coupling agents (e.g., PyBOP) has been utilized for ligating N- and C-termini via amide bond formation in SFTI-1 and kB1 peptide synthesis.^16−18^

In the current work, the production of cyclic peptides SFTI-1 and kB1 mediated by recombinant PatGmac macrocyclase is described. Linear precursor variants of SFTI-1 and kB1 were synthesized by Fmoc SPPS comprising the enzyme recognition sequence. PatGmac macrocyclization activity was then monitored by a combination of LC-MS and NMR.

## Results and Discussion

Linear SFTI-1 and kB1 precursors with the PatG recognition motif (-AYDG) at the C-terminus were assembled using a combination of automated and manual Fmoc chemistry. Peptides were cleaved from the resin and then purified using RP-HPLC. Mass spectrometry was used to confirm the identity of the products. In total, five precursor peptides were synthesized based on SFTI-1 and kB1 as illustrated in Table 1.

**Table 1:**
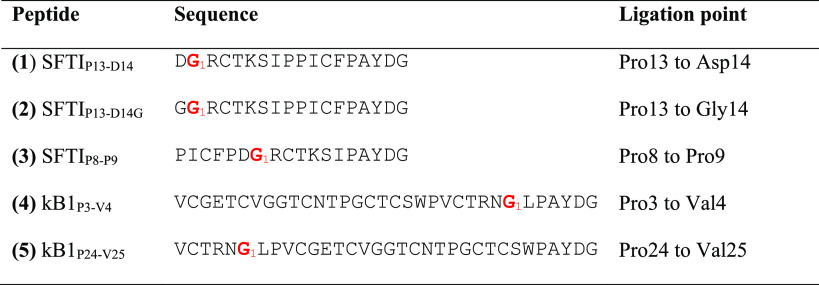
Peptide Sequences and Ligation Points^a^

a Numbering of residues and ligation points is based on the genetic sequences of peptides. The first residue is highlighted in red.

**Figure 1:**
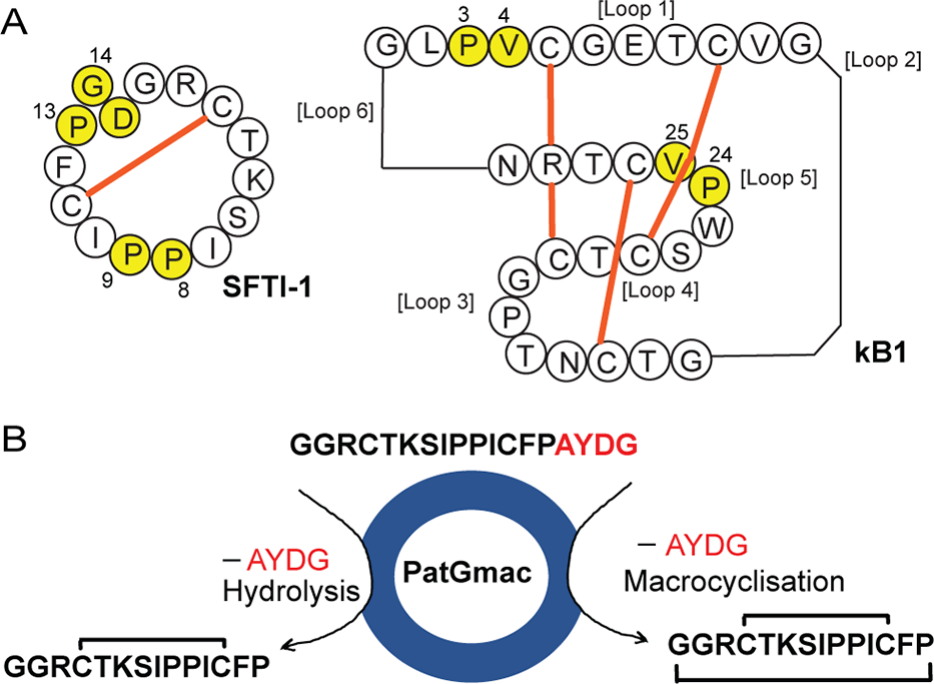
Peptide ligation sites and macrocyclization by PatGmac. (A) Schematic illustrations of SFTI-1 and kB1 sequences with the ligation sites used in the current work (highlighted in yellow). Note the disulfide bonds between Cys (C) residues. (B) Schematic illustration of PatGmac-mediated cyclization. PatGmac requires a C-terminal proline residue and the sequence motif AYDG at sites P1′–P4′. The substrate, here SFTI_P13-D14G_, is processed to give the macrocyclic peptide minus the AYDG motif, or a linear product. The reaction can be monitored by the difference in molecular weights as cyclization confers loss of water (−18 Da). In the current work, fully reduced peptides were used as initial substrate, but oxidation of disulfide bonds occurs during cyclization.

Precursor peptides were made to contain a Pro residue preceding the macrocyclization AYDG motif at the P1′–P4′ position. The AYDG motif is subsequently cleaved off to form the head-to-tail cyclized peptide as shown in Figure 1. SFTI-1 contains three Pro residues and thus three possible ligation sites (i*.*e., with Pro in the P1 position). One Pro is proceeded by Asp, which has an acidic side chain. Hence, we decided to make a peptide with that residue substituted with a Gly. These peptides are **1** and **2**, as shown in Table 1. Then, because SFTI-1 contains two Pro residues in sequence, we choose to exclude them to make the peptide with Pro at both P1 and P2, making only **3** that has Pro as P1 and P1′.

From previous experience of the synthesis of similar peptides in our laboratory, aspartimide formation has been an obstacle in Fmoc-based-SPPS. This reaction is sequence-dependent and results in ring closure between the N of the α-carboxy amide bond and the β-carboxy side chain of Asp, due to repeated exposure to the piperidine base during Fmoc deprotection.^19^ In the synthesis of **1** and **2**, 5% piperazine in DMF containing 0.1 M HOBT^20^ was used as the deprotecting agent for both manual and microwave-assisted automated synthesis, to minimize aspartimide formation. Under the above condition, no aspartimide side products were observed. During the course of the current work, HOBT was phased out as a coupling agent in SPPS. Thus, we examined Oxyma pure^21^ as a more safe, stable, and inexpensive alternative in place of HOBT. This resulted in successful manual synthesis of both **3** and **5**. Precursor **4** was assembled using 5% piperazine as a deprotecting agent and a dipeptide, Fmoc-Asp(OtBu)-(Dmb)Gly-OH, to prevent aspartimide formation.^22^

**Figure 2:**
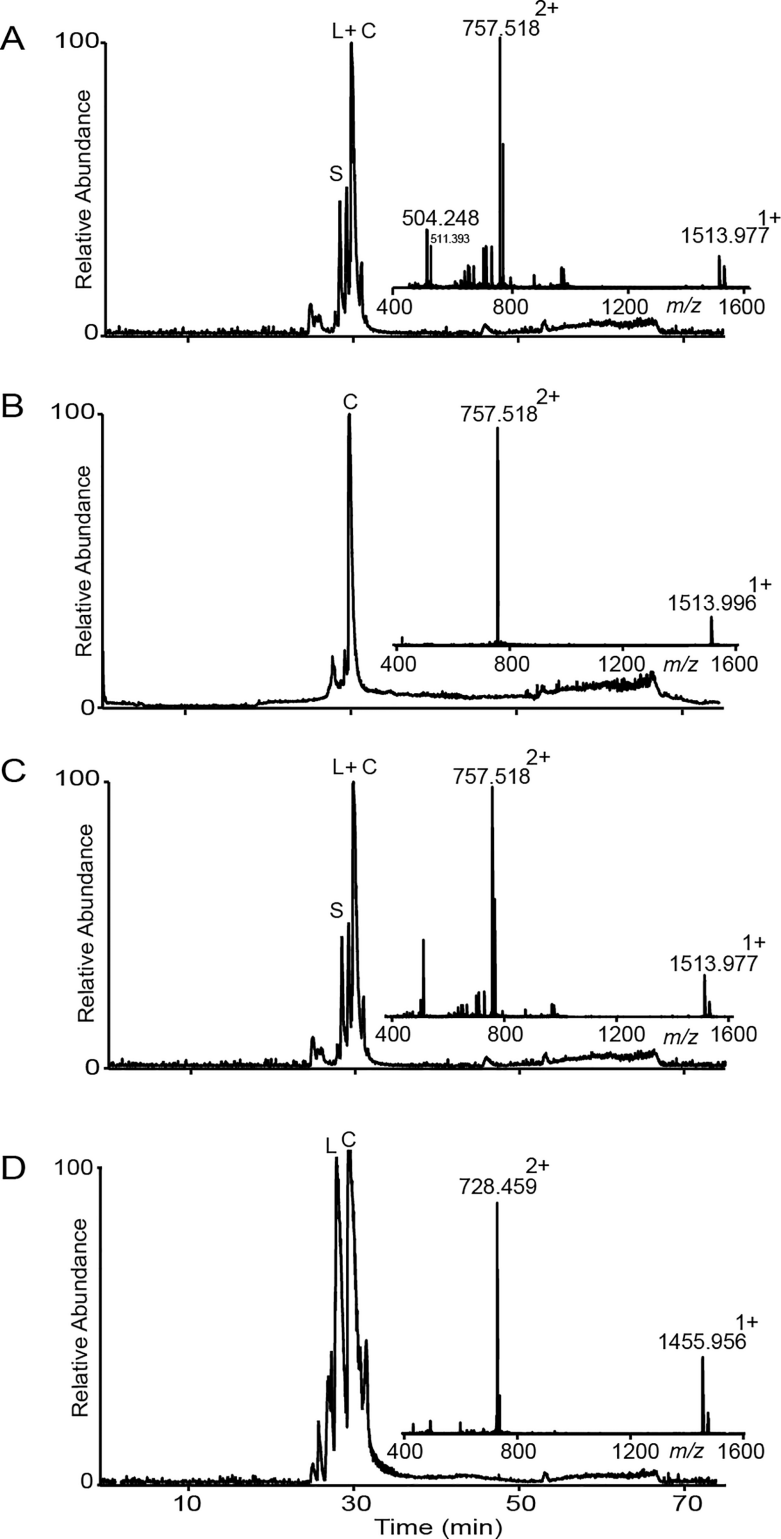
LC-MS characterization of PatGmac-mediated backbone cyclization of SFTI-1 peptides. (A) Substrate **1** (marked S) gives both linear (L) and cyclic peptide (marked C). L and C practically coelute. Mass spectra of cyclic **1** are inserted, showing the monoisotopic mass 1513.97 (calculated monoisotopic mass 1513.81). (B) Native SFTI-1 isolated from sunflower seeds with an observed mass of 1513.99. (C) Co-injection of enzyme cyclized **1** and native SFTI-1. (D) No substrate was detected for **2**, only linear and cyclic products. Note that these peptides show good separation. The inserted spectra show the monoisotopic mass 1455.95 for C (calculated monoisotopic mass 1455.77).

Macrocyclization were carried out with 150 μM of peptide and 35 μM of PatGmac at pH 7.5 over a time of 120 h. Incubation time and concentrations are similar to previous cyclization of smaller peptides, including patellamides.^4^ From the start, peptides were all in reduced form, without disulfides. At these buffer conditions, oxidation occurs spontaneously. After incubation, reaction mixtures were analyzed using LC-MS and LC-UV. The results for SFTI-1 peptides are shown in Figure 2. Cyclic **1** (experimental monoisotopic mass 1513.97, calculated monoisotopic mass 1513.81) and acyclic **1** (exptl 1531.96, calcd 1531.81) were detected as shown in Figure 2A. Upon co-injection, PatGmac cyclized **1**, and native SFTI-1 (isolated from sunflower seeds) eluted in one chromatographic peak, confirming identical structures (Figure 2B and C). Similarly, analysis of the reaction mixture of **2** revealed masses for cyclic **2** (exptl 1455.95, calcd 1455.77) and acyclic **2** (exptl 1474.04, calcd 1473.77) as shown in Figure 2D.

**Figure 3:**
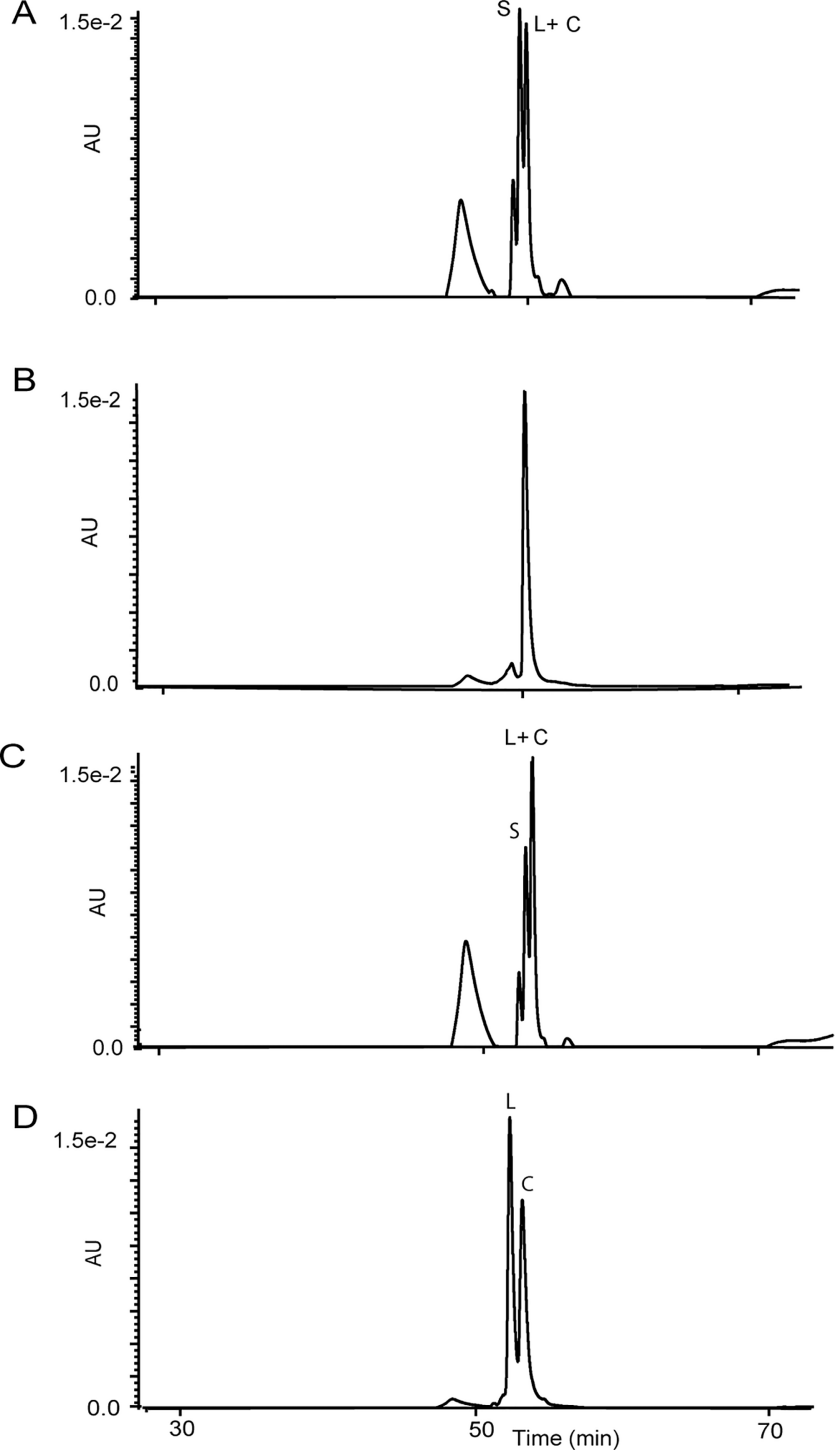
LC-UV profile of PatGmac cyclized SFTI-1 peptides. (A) The substrate **1** (marked S) gives both linear and cyclic peptide, marked L and C. The latter practically coelute. (B) Native peptide isolated from sunflower seeds (C). (C) The reaction mixture from enzyme cyclized **1** and native peptide coelute. (D) Linear and cyclic **2** separate well, and cyclic **2** is obtained at a yield of 45% as calculated from absorbance at 215 nm.

For **1**, the linear hydrolyzed peptide and the cyclic product practically coeluted, making it difficult to determine a yield. For peptide **2**, containing the Asp/Gly substitution, cyclic and linear peptides eluted with near-baseline separation. The yield of cyclic **2** was calculated to be 45%, as judged by integration after LC-UV analysis as shown in Figure 3D. Analysis of the reaction mixture of **3** showed only masses for acyclic linear **3** and the starting material (data not shown) and no cyclic product. Hence, the two sequential prolines inhibit cyclization. There was no cyclic product detected in control samples, indicating that no spontaneous cyclization occurred independent of the enzyme.

**Figure 4:**
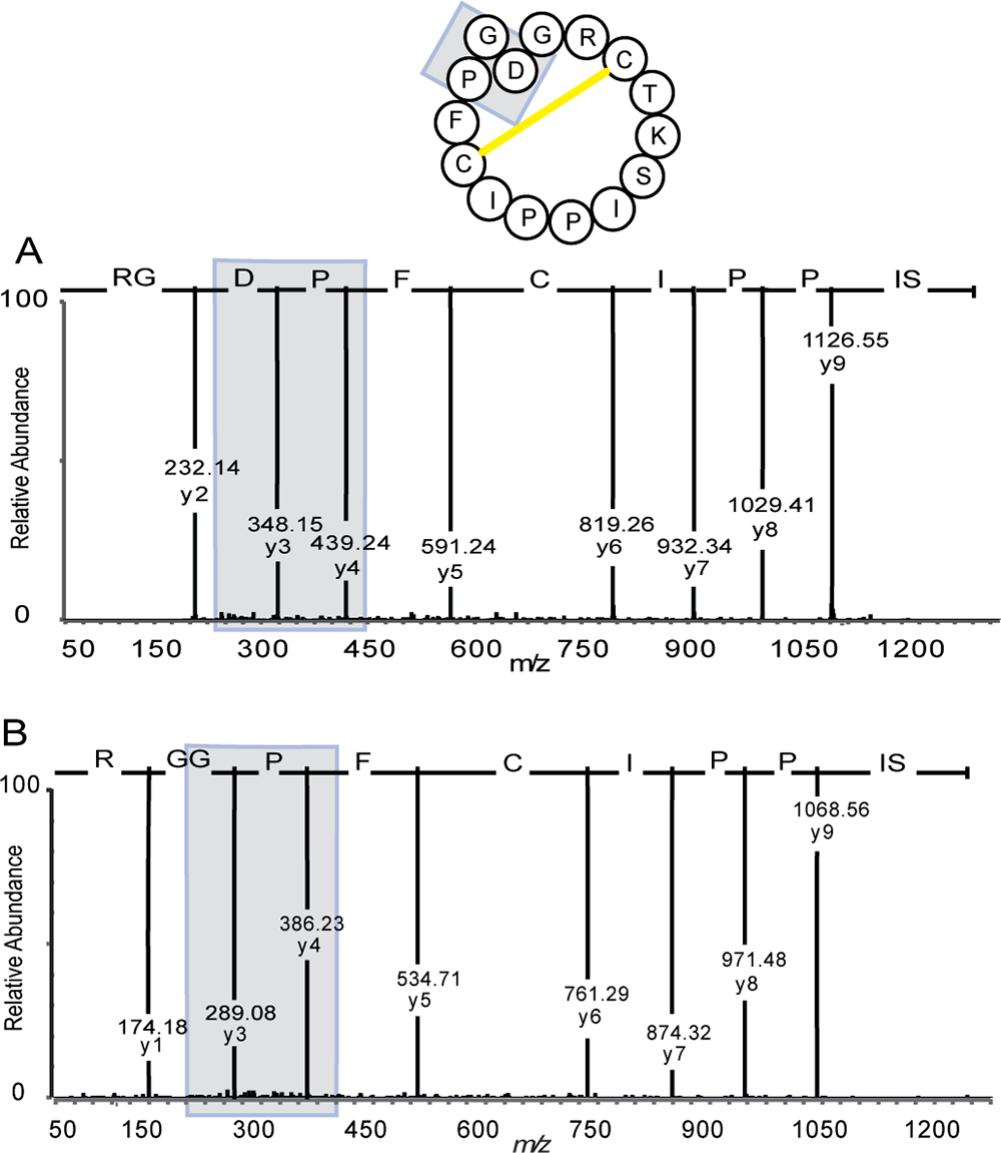
LC-MSMS spectra for tryptic fragments of SFTI peptides: The backbone cyclization of SFTI-1 peptides is demonstrated by identification of peptide fragments overlapping the ligation site. (A) Reduced, alkylated, and trypsin cleaved enzyme cyclized **1** fragment (SIPPICFPDGR) with a mass of 1326.66 Da spans the amide bond at the ligation site between P13 and D14. (B) Tryptic cleaved enzyme cyclized **2** fragment (SIPPICFPGGR) with a mass of 1268.62 Da spans the amide bond at the ligation site between P13 and D14G.

MS was then used to sequence cyclic peptides and prove the ligation point. To make the current peptides amenable for sequencing, disulfides must first be reduced and alkylated and the cyclic peptide cleaved into a linear one. Here, disulfide bonds were reduced with tris(2-carboxyethyl)phosphine and alkylated with *N*-ethylmaleimide, before being digested by trypsin. The obtained linear peptide chain was then subjected to MSMS. For cyclic **1**, a fragment with a mass of 1326.66 Da was identified corresponding to SIPPICFPDGR, resulting from trypsin cleavage after R2 and K5. This fragment spans the amide bond at the ligation site between P13 and D14. The cyclic backbone and point of ligation were unequivocally confirmed by MSMS fragmentation as shown in Figure 4A. Following trypsin cleavage of cyclic **2**, a fragment corresponding to SIPPICFPGGR (1268.62 Da) was identified, resulting from cleavage after R2 and K5. As above, this fragment confirmed the cyclic backbone because it spans the amide bond at the ligation site between P13 and G14. The MSMS fragmentation is shown in Figure 4B.

**Figure 5:**
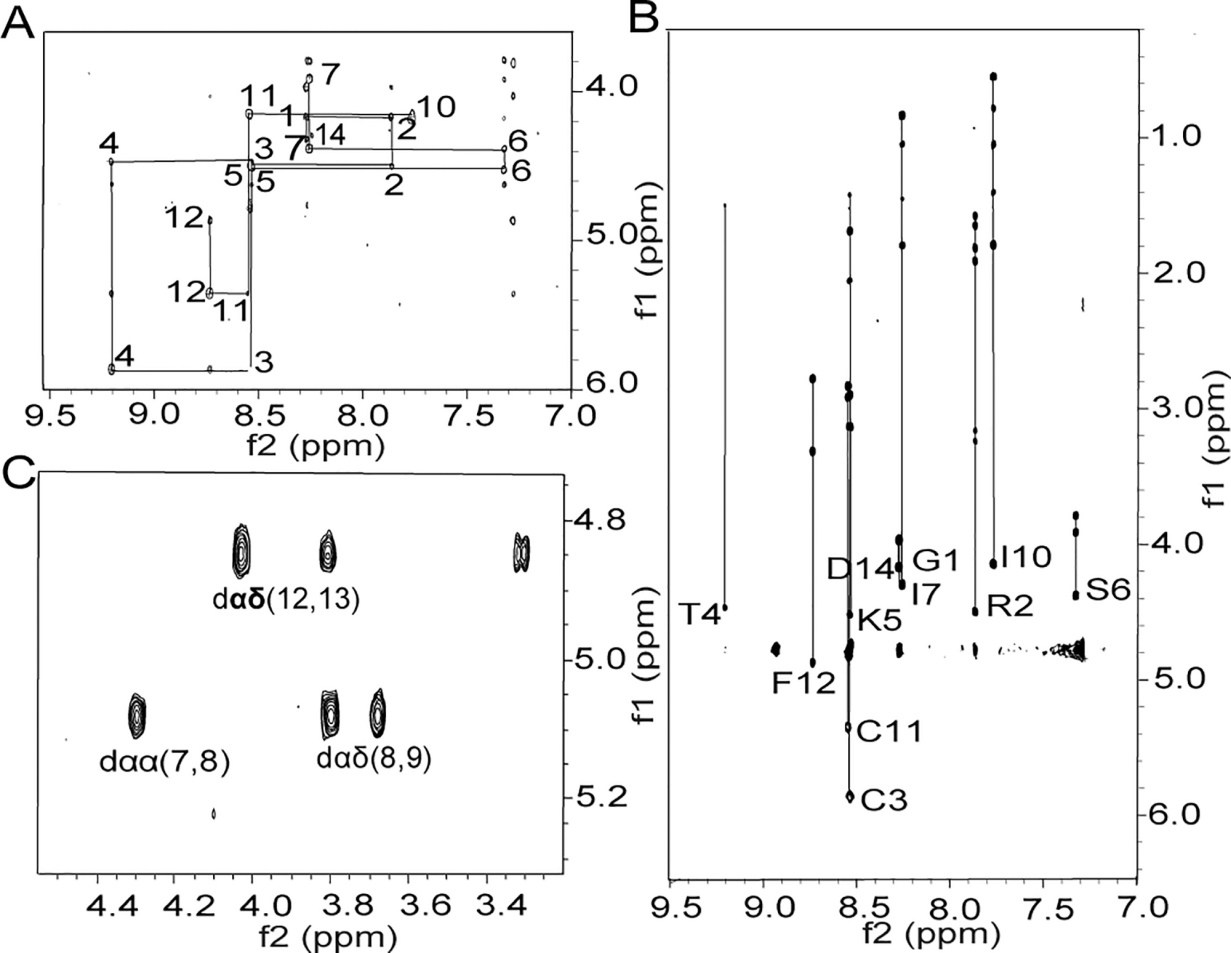
NMR analysis of cyclic 2, SFTI_P13-D14G_. (A) NH-H^α^ fingerprint region of the NOESY spectrum of **2** showing intraresidue connectivities. (B) Sequential *d*_αN(*i*,*i*+1)_ connectivities in the NH-H^α^ fingerprint region of the TOCSY spectrum. The sequential connectivity pattern is only broken at the proline residues between I7 and I10 and between F12 and D14. (C) H^α^–H^α^ region of the NOESY spectrum. The *d*_αα(*i*-1,*i*)_ and *d*_αδ(*i*-1,*i*)_ connectivities of prolines.

The native fold of cyclic **1** was supported by coelution with isolated SFTI-1, but no such reference exists for cyclic **2**. Hence, to confirm the structure of this peptide, we used NMR. Signals of cyclic **2** were well dispersed in the amide proton region, indicating that the peptide is folded and has a well-defined structure (secondary chemical shifts are shown in Supplementary Figure S1.). The spin systems of all individual amino acids were assigned using TOCSY and NOESY experiments, as shown in Figure 5, together with sequential assignments in the fingerprint region. αH-HN sequential connectivity is unbroken apart from the Pro residues. Strong NOEs for *d*_αα(7_,_8)_ confirmed the presence of a *cis* amide bond preceding P8. On the contrary, strong NOEs for *d*_αδ (8_,_9)_*and d*_αδ (12_,_13)_confirmed the presence of *trans* amide bonds preceding P9 and P13. Thus, the individual Pro conformations are identical to those in the native peptide and provide evidence that cyclic **2** has a native conformation.

**Figure 6:**
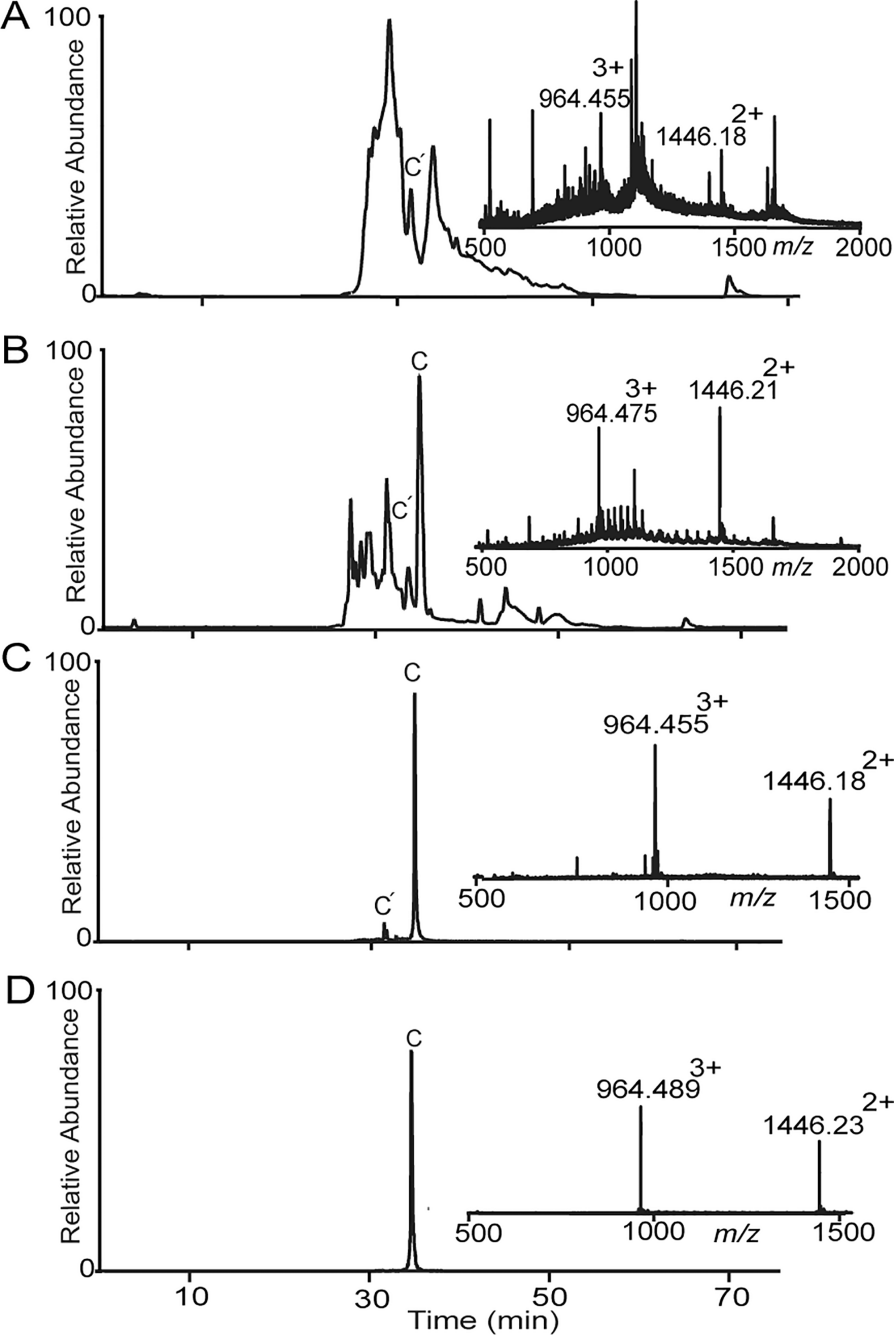
LC-MS for PatGmac cyclized kB1 peptides. (A) Enzyme cyclized **4** (observed monoisotopic mass 2891.36 (M + H^+^) and calculated monoisotopic mass 2891.34). (B) Co-injection of enzyme cyclized **5** and native kB1 eluted separately, suggesting different disulfide connectivity. (C) Cyclic **4** and native kB1 co-injection. (D) Native kB1 peptide isolated from *O. affinis*.

Two kB1 precursor peptides with two different ligation sites were synthesized and subjected to PatG macrocyclase cyclization using the same buffer conditions and reaction time as above. For peptide **4**, the ligation site was between P3 and V4 in loop 6, and for peptide **5** between P24 and V25 in loop 5. Reaction mixtures were analyzed using LC-MS and compared with native kB1 peptide as isolated from the plant *Oldenlandia affinis*. PatGmac cyclized kB1 peptides were obtained with a mass corresponding to native kB1 as shown in Figure 6 but with a different retention time. This indicates that these peptides contain non-native disulfide connectivity. The overall yield of the cyclic product was low for both kB1 peptides.

**Figure 7:**
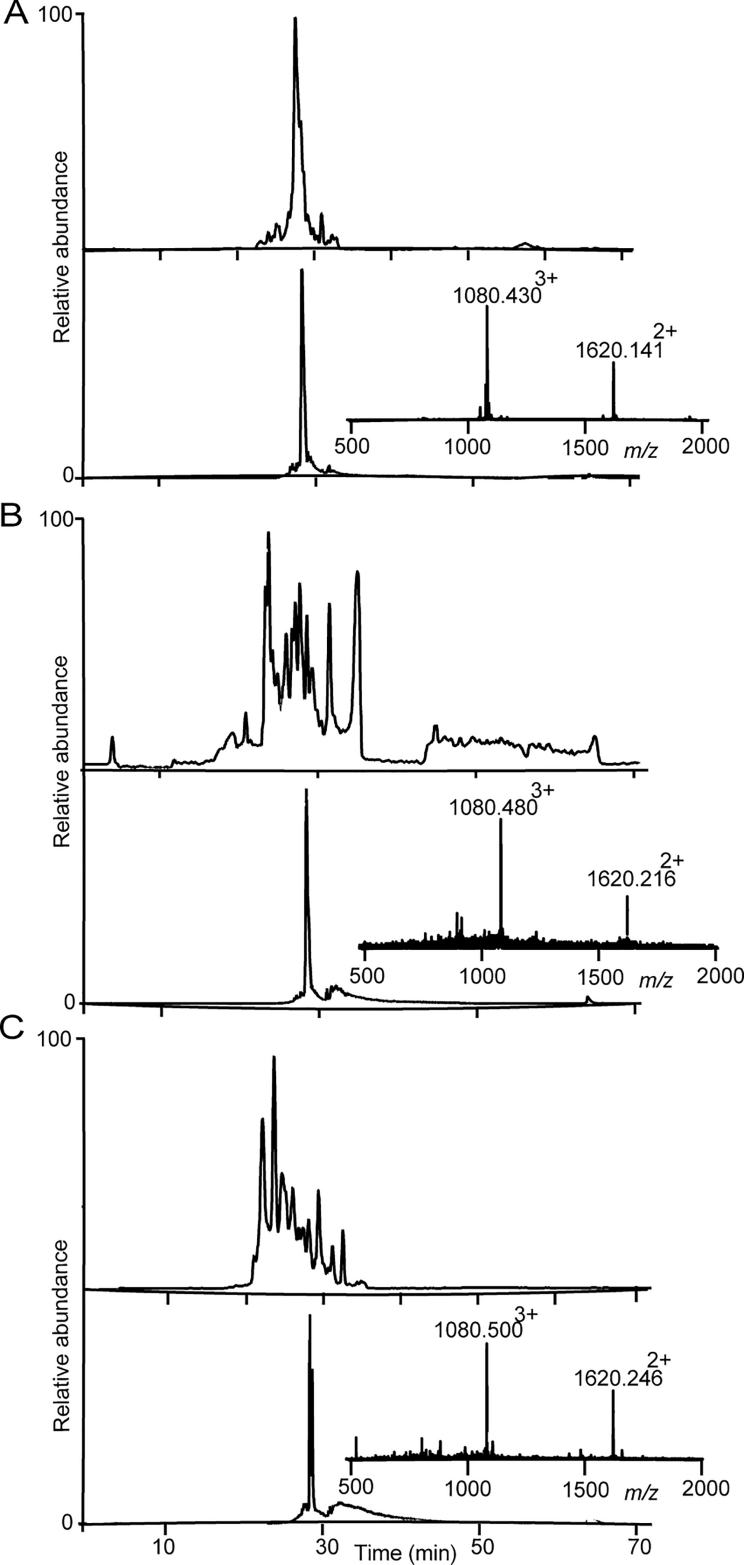
LC-MS for cyclic and alkylated kB1 peptides. Peptides containing three disulfide bonds can theoretically form 15 different isomers. Hence, disulfide bonds were reduced and alkylated to facilitate identification of the cyclic products, which then elute in one single peak. This procedure adds 348 mass units (+58 for each cysteine involved in a disulfide bond). Chromatograms and mass spectra show the alkylated peptides on LC-MS. (A) Reduced and alkylated native kB1. At the top, the base peak ion chromatogram and below the extracted ion chromatogram for the doubly charged ion. Inserted is the mass spectrum. (B) Reduced and alkylated **4**, showing a molecular weight of 3239.42 (M + H^+^). The peptide has gained 348.0 Da, demonstrating that all six cysteines are alkylated. (C) Reduced and alkylated **5**.

To confirm that the difference was only in disulfide connectivity, the two cyclic peptides were reduced, alkylated, and again compared to reference kB1 (i.e., peptide isolated from the plant that was also reduced and alkylated). Under these conditions, retention times were identical for all peptides, and observed masses were also similar, as shown in Figure 7A–C.

In the current work, we explored the limits of PatG macrocyclase for the production of cyclic disulfide-rich peptides using two prototypic peptides of 14 and 29 amino acids, respectively. We found that PatGmac can be used for the production of both cyclic peptides, but also that yield decreased for the larger kalata B1. Using PatGmac, **1** and **2** were cyclized, and then oxidized correctly, in a one-pot step. LC-MS analysis confirmed the presence of both cyclic and hydrolyzed linear **1** and **2**. The elution pattern of the cyclized **1** was identical to the native peptide isolated from sunflower seeds. For **2**, NMR confirmed the native fold, including identical proline conformations to that in the native SFTI-1. The P1 Pro in patellamides are all in *cis* conformation, and it has been reported that this is required for the efficient binding of the substrate in the enzyme-binding pocket.^4^ In contrast, in native SFTI-1 Pro13 is in *trans* conformation,^23^ and it is evident from the current study that this does not hinder cyclization.

LC-MS analysis of the PatGmac cyclized kB1 peptides confirmed the removal of the recognition tag (-AYDG) and a loss of H_2_O, indicative of head-to-tail cyclization. However, none of the PatGmac cyclized kB1 peptides coeluted with native kB1, indicating a non-native disulfide connectivity despite the formation of the cyclic backbone. Notably, when kB1 peptides were reduced and alkylated, they coeluted with native kB1, confirming identical structures, as shown in Figure 6. Thus, our findings show that production of kB1 peptides with native disulfide connectivity was not possible, at least not with the current reaction conditions (500 mM NaCl, 10 mM bicine, 5% DMSO at pH 7.5) used for PatGmac-mediated cyclization. Previously, Aboye et al. reported the use of DMSO-containing buffer for the oxidative folding of kB1 resulting in poor yield of native disulfide fold.^24^

In contrast to SFTI-1 peptides, the PatGmac macrocyclization was not as efficient for kB1, presumably due to its large size and multiple disulfide bonds. It is reported for sortase A (SortA) cyclization of kB1 that no correctly folded peptide was produced when oxidation occurred after cyclization, but correctly folded peptide was produced when oxidation occurred prior to backbone cyclization with resulting higher yields.^25^ Most likely, there is a room to address the low yield in kB1 peptides by modifying the reaction conditions or employing an approach where oxidation occurs prior to cyclization.

The presence of Pro residues in SFTI-1 and kB1 makes these good models to study cyclization with PatGmac. Our results confirm that it is possible to use this method for the cyclization of peptides in sizes up to 29 residues, although with yields diminishing with size. One of the limitations of the PatGmac method was the requirement for a thiazoline/l-Pro at the C-terminus of the recognition tag. Recently, Queis and co-workers addressed the above problem and showed that the PatGmac method can be used to cyclize peptides containing non-natural and natural proline mimics at the C-terminus of the recognition tag with moderate yields (32–40%).^26^ The macrocyclization method using PatG can also be extended to a wide range of other disulfide-rich peptides (e.g., conotoxins^27^ and defensins^28^) and macrocyclic antimicrobial peptides^29,30^ with therapeutic potential.

The main advantages of cyclic peptides are their remarkable biological and chemical stability. The peptides in focus in the current work are of interest for the development of therapeutics and for use as scaffolds to carry pharmaceutically active epitopes.^2,31,32^ However, the main obstacles in the development of these peptides as potential therapeutics are their backbone cyclization and oxidative folding. Currently, several methods for the chemical and biological synthesis of natural and engineered cyclotides and SFTI-1 have been reported, most of which include native chemical ligation as the last step.^33^ However, enzymes that do not have cyclization functions in nature such as the bacterial transpeptidase enzyme sortase A and inteins have also been utilized to produce a wide range of cyclic peptides including kB1 and SFTI-1. Although sortase A was successful in the production of cyclic peptides, four additional non-native residues that are remnants of the C-terminal recognition sequence (LPXT) get incorporated within the cyclized backbone.^25^ A modified version of subtilisin from *B. subtilis*, omniligase-1, has also been reported to cyclize cyclotide kB1 and MCoT I-II, with the advantage that no “footprint” recognition sequence remains in the cyclized peptide upon cyclization.^34,35^ Other proteases, e.g., trypsin, have been also employed for the back cyclization of MCoTI-II and SFTI-1.^36,37^ The expressed protein ligation (EPL) technique has also been used for the production of disulfide-rich backbone cyclized peptides, including SFTI-1 and kB1.^38,39^

To date, a handful of naturally occurring cyclases have been characterized in detail, including peptide cyclase 1 (PCY1), prolyl oligopeptidase (POP), and two asparaginyl endopeptidases, i.e., butelase 1 and *Oa*AEP1.^40−44^ PCY1 is involved in the biosynthesis of Caryophyllaceae-type cyclic peptides or orbitides; POPB is a cyclase from the family of serine proteases, catalyzing the cyclization of amatoxins;^41,45^ and butelase 1 and *Oa*AEP1 are both cyclotide cyclizing enzymes, which have been reported to cyclize a wide range of substrates of varying length efficiently.^29,44,46,47^ To date, butelase appears as the most versatile enzyme, but obtaining this enzyme in large amounts has been elusive. In contrast, PatGmac is straightforward to produce by recombinant expression.

## Experimental Section

### Reagents

All Fmoc-protected amino acids were purchased from ChemPep Inc. or Iris Biotech GmbH. The following resins were used: Fmoc-Tentgel R Ram Rink-type resin (0.18 mmol/g, Peptide International, Fmoc-Gly-Novasyn TGT (0.2 mmol/g), Merck KGaA and Fmoc-Gly(Dmb)-Tentagel (0.18 mmol/g), Rapp Polymere GmbH. Di-Fmoc-3,4-diaminobenzoic acid was from AnaSpec. 2-(1*H*-Benzotriazole-1-yl)-1,1,3,3-tetramethyluronium hexafluorophosphate) (HBTU) was purchased from PepChem. *N*,*N*-Dimethylformamide (DMF) was from Honeywell Burdick & Jackson. HPLC-grade acetonitrile (MeCN) was from VWR Chemicals. Diethyl ether was purchased from Merck KGaA. Diisopropylethylamine (DIPEA), triisopropylsilane (TIPS), piperidine, piperazine, ethyl(hydroxyamino) cyanoacetate (Oxyma pure), 1-hydroxybenzotriazole hydrate (HOBT), trifluoroacetic acid (TFA), tris(2-carboxyethyl)phosphine hydrochloride (TCEP·HCl), *N*-ethylmaleimide (NEM), and iodoacetamide (IAM) were from Sigma-Aldrich. PD-10 desalting columns (Sephadex G-25 resin) for size exclusion chromatography were from GE Healthcare Bio-Sciences.

### Peptide Synthesis

Peptides **1** and **2** were synthesized on Fmoc-Gly-Novasyn TGT resin at a 0.1 mm scale synthesis. Deprotection of the Fmoc group was carried out in 5% piperazine in DMF containing 0.1 M HOBT. The first four residues were coupled manually, and the rest of the sequence was elongated by a microwave-assisted Fmoc/HBTU-SPPS protocol (MW-assisted deprotection at 75 °C, 3 min, and MW-assisted coupling at 75 °C, 5 min) on a Liberty1 microwave peptide synthesizer (CEM Corp.). Peptides **3** and **5** were assembled by manual Fmoc-SPPS on a Novasyn TGT(Gly) resin using a 0.1 mm scale. Deprotection was done using 20% piperidine supplemented with 1 M Oxyma pure. Each amino acid residue (4 equiv) was coupled *in situ* with HBTU (4 equiv) and DIPEA (6 equiv) for 30 min.

After completion of synthesis, resin-bound peptides were cleaved off from the resin using a strong cleavage mixture containing TFA, TIPS, and H_2_O (95:2.5:2.5) at room temperature (rt) for 90 min. TFA was reduced with a flow of N_2_, and the peptide was precipitated with ice-cold diethyl ether. The collected precipitate was dissolved in 50% MeCN containing 0.05% TFA and lyophilized.

Peptide **4** was assembled on a Tentagel Rink amide resin (0.19 mmol/g) on a 0.1 mm scale, using an optimized protocol for microwave-assisted synthesis. The C-terminal di-Fmoc-3,4 diaminobenzoic acid (Dbz group) and the first three residues were coupled manually. The rest of the peptide was elongated by microwave-assisted automated synthesis. Following the completion of the linear chain, the C-terminal Dbz group was acetylated using *p*-nitrophenylchloroformate and thus activated by DIPEA to yield the resin-bound *N*-acyl urea peptide (Nbz). The Nbz peptide was then fully deprotected and cleaved from the resin by strong cleavage. Cleaved freeze-dried Nbz peptide was hydrolyzed and converted into linear peptide in NH_4_HCO_3_ buffer (pH 8.5, overnight).

### PatG Cyclization

Macrocyclization reactions were prepared for 150 μM peptide substrate and 35 μM enzyme in a buffer containing 500 mM NaCl, 10 mM bicine pH 7.5, and 5% DMSO. Reaction mixtures were incubated for 120 h at 30 °C. Samples were analyzed by LC-UV-MS. Control samples were prepared by incubation of the peptide substrates in the aforementioned buffer.

### Reduction and Alkylation

Lyophilized desalted samples were dissolved in 100 μL of TCEP buffer (1 mM TCEP·HCl in 50 mM NH_4_HCO_3_, pH 8.5) and incubated at 65 °C for 10 min. The reduced SFTI peptides were subsequently alkylated with 60 mM NEM in 0.2 M citrate buffer at pH 3 (37 °C, 2 h), and kB1 peptides were alkylated with IAM (50 mg, in 0.5 M Tris-HCl, 2 mM EDTA). After incubation for 10 min at rt, the reaction was terminated by adding 5 μL of TFA. The alkylated peptides were purified using gel filtration (PD 10) using 30% MeCN and 0.1% TFA in H_2_O as the mobile phase. The alkylated SFTI peptides were digested with trypsin dissolved (sequencing grade, Promega Co.) in 50 mM NH_4_HCO_3_ (pH 7.8, 37 °C, 4 h) and analyzed by LC-MSMS.

### HPLC

Peptides were purified with RP-HPLC on a Shimadzu LC10 AD (Shimadzu, Japan) with 215, 254, and 280 nm detection. Preparative HPLC was performed using a Phenomenex Jupiter C18 column (250 × 10 mm, 5 μm), and analytical HPLC was performed on a Phenomenex Jupiter C18 column (250 × 4.6 mm, 5 μm) at a flow rate of 1 mL/min. Solvents A (10% MeCN, 0.05% TFA in H_2_O) and B (60% MeCN, 0.05% TFA in H_2_O) were used in a linear gradient from of 0 to 80% solvent B over 70 min for the preparative and analytical HPLC.

### LC-UV-MS/MSMS

The cyclization products as well as reduced and alkylated peptides were analyzed using a UPLC-Qtof nanospray MS (Waters nanoAcquity, 75 μm × 250 mm 1.7 μm, BEH130 C18) coupled to a QToF Micro. The scan window was set to 200–2000 *m*/*z* and for MSMS to 50–2000 *m*/*z*. A linear gradient from 0 to 70% solvent D (0.1% formic acid (FA) in MeCN) in solvent C (0.1% FA in H_2_O) over 70 min at a flow rate of 0.3 μL/min was used for analysis.

### NMR

Freeze-dried peptide (1.2 mg) was dissolved in 600 μL of H_2_O/D_2_O (9:1, v/v) at pH ∼5. Two-dimensional spectra were recorded at 298 K on a Bruker 900 MHz spectrometer equipped with a cryogenic probe. All data, including 1D, TOCSY (mixing time 80 ms), and NOESY (mixing time 150 ms) were recorded and processed using Topspin (Bruker). The water signal was suppressed using a modified WATERGATE sequence. Generally, 4096 data points were collected in the F2 dimension and 256 (128 complex) points in F1, with 512 increments of 8 scans over 1194 Hz.

## Supporting Information Available

Comparison of secondary chemical shifts of cyclic product **2** and native SFTI-1 (PDF)

## Dedication

Dedicated to Dr. Mary J. Garson, The University of Queensland, for her pioneering work on bioactive natural products.
