# Supplementary material for: Exploring the Limits of Cyanobactin Macrocyclase PatGmac: Cyclization of PawS-Derived Peptide Sunflower Trypsin Inhibitor-1 and Cyclotide Kalata B1
Source: J Nat Prod. 2023 Mar 14;86(3):566–73. doi: 10.1021/acs.jnatprod.2c01158 (PMC10043927; doi:10.1021/acs.jnatprod.2c01158)
Supplement: Supplementary file 1 — np2c01158_si_001.pdf [file np2c01158_si_001.pdf]

Exploring the Limits of Cyanobactin Macrocyclase PatGmac: Cyclisation of PawS Derived Peptide Sunflower Trypsin Inhibitor-1 and Cyclotide Kalata B1

Taj Muhammad[1], *Wael E Houssen*[2,3], *Louise Thomas*[2,3], *Cristina-Nicoleta Alexandru-Crivac*[2,3,4], *Sunithi Gunasekera*[1], *Marcel Jaspars* [2], *Ulf Göransson* [1 *]

[1]Pharmacognosy, Department of Pharmaceutical Biosciences, Uppsala University, Biomedical Centre, Box 591, SE-75124 Uppsala, Sweden

[2]Department of Chemistry, Marine Biodiscovery Centre, University of Aberdeen, Aberdeen AB24 3UE, Scotland, UK.

[3]Institute of Medical Sciences, University of Aberdeen, Aberdeen AB25 2ZD, Scotland, UK.

[4]Current address: The University of Sheffield, Chemical and Biological Engineering Department, Sir Robert Hadfield Building, Mappin St, Sheffield S1 3JD

Table of content

Item	Page number
**Figure S1.** Secondary Chemical shift	3

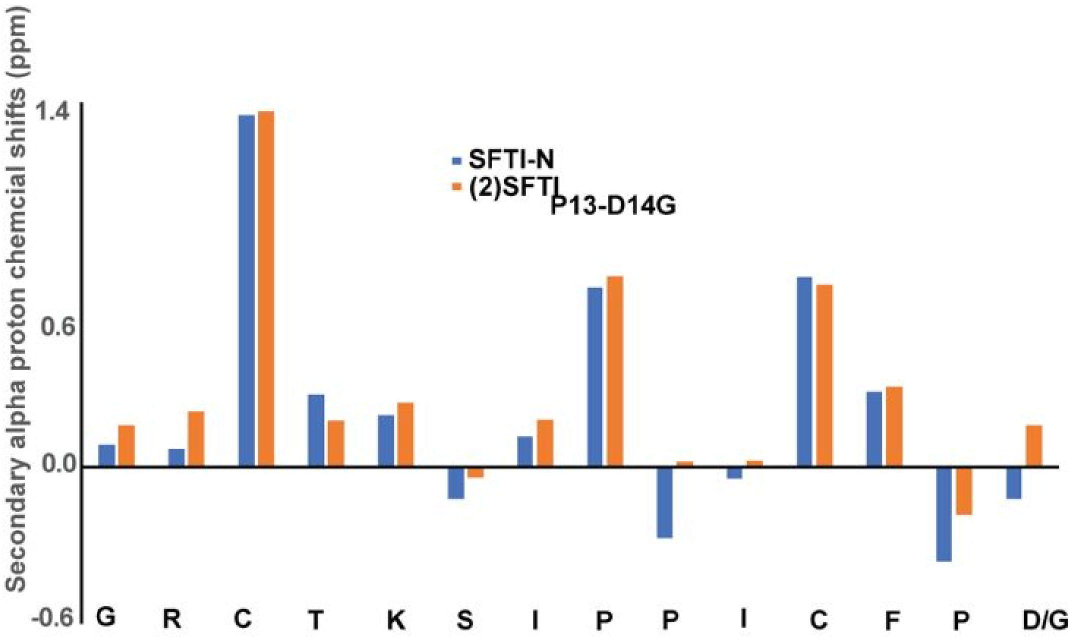

Figure S1: Comparison of secondary chemical shifts of cyclic 2 SFTI$_{P13\text{-}D14G}$ with native SFTI-1. Secondary chemical shift was calculated by subtracting the random coil of αH chemical shift from the experimental αH chemical shift. The blue bars represent cyclic native SFTI-1 and the orange bars represent cyclic **2**.
